# Design and simulation of type-I graphene/Si quantum dot superlattice for intermediate-band solar cell applications

**DOI:** 10.1007/s12200-022-00043-2

**Published:** 2022-10-28

**Authors:** Masumeh Sarkhoush, Hassan Rasooli Saghai, Hadi Soofi

**Affiliations:** 1 Department of Electrical Engineering, Shabestar Branch, Islamic Azad University, Shabestar, 5381637181 Iran; 2 Department of Electrical Engineering, Tabriz Branch, Islamic Azad University, Tabriz, 5167636137 Iran; 3 Faculty of Electrical and Computer Engineering, University of Tabriz, Tabriz, 5166616471 Iran

**Keywords:** Graphene/silicon quantum dot, Intermediate-band, Solar cell, Superlattice

## Abstract

Recent experiments suggest graphene-based materials as candidates for use in future electronic and optoelectronic devices. In this study, we propose a new multilayer quantum dot (QD) superlattice (SL) structure with graphene as the core and silicon (Si) as the shell of QD. The Slater–Koster tight-binding method based on Bloch theory is exploited to investigate the band structure and energy states of the graphene/Si QD. Results reveal that the graphene/Si QD is a type-I QD and the ground state is 0.6 eV above the valance band. The results also suggest that the graphene/Si QD can be potentially used to create a sub-bandgap in all Si-based intermediate-band solar cells (IBSC). The energy level hybridization in a SL of graphene/Si QDs is investigated and it is observed that the mini-band formation is under the influence of inter-dot spacing among QDs. To evaluate the impact of the graphene/Si QD SL on the performance of Si-based solar cells, we design an IBSC based on the graphene/Si QD (QDIBSC) and calculate its short-circuit current density (*J*_sc_) and carrier generation rate (*G*) using the 2D finite difference time domain (FDTD) method. In comparison with the standard Si-based solar cell which records *J*_sc_ = 16.9067 mA/cm^2^ and *G* = 1.48943 × 10^28^ m^−3^⋅s^−1^, the graphene/Si QD IBSC with 2 layers of QDs presents *J*_sc_ = 36.4193 mA/cm^2^ and *G* = 7.94192 × 10^28^ m^−3^⋅s^−1^, offering considerable improvement. Finally, the effects of the number of QD layers (*L*) and the height of QD (*H*) on the performance of the graphene/Si QD IBSC are discussed.

**Graphical abstract:**

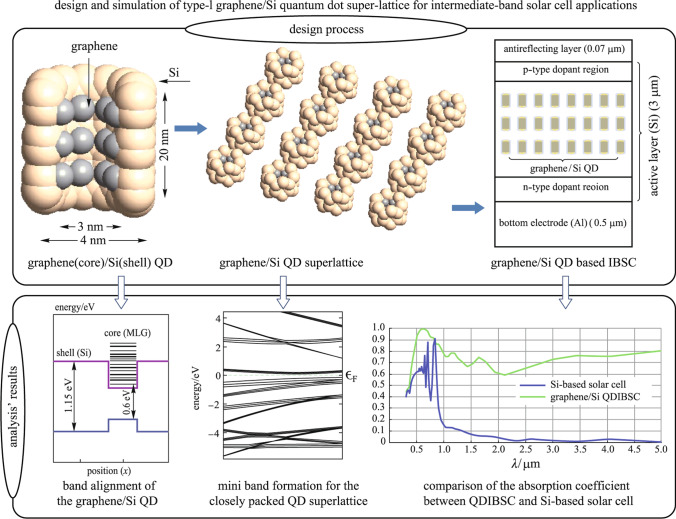

## Introduction

The intermediate-band solar cell (IBSC) proposed by Luque and Marti is a concept that allows the Shockley–Queisser limitation to be overcome [1]. In this concept, an energy band is introduced within the semiconductor material bandgap of the active layer called the intermediate-band (IB). This IB provides a potential for sequential absorption of photons with sub-bandgap energy that would otherwise be transmitted. In the past few years, various techniques have been proposed to incorporate the basic principles of IBSCs. The most efficient approach is by employing quantum dots (QDs) [2]. The IB is introduced by confining the electrons in a QD. It was anticipated that the conversion efficiency of IBSC based on QD (QDIBSC) would rise to about 45% under light intensity of one sun and 63% under concentrated illumination for an ideal single junction cell [1]. Indeed in recent years, multilayer QDs have gained increasing research interest for IBSC applications. By tuning the properties of a multilayer QD, such as dimensions, shape, and materials of the core and shell layers, QDIBSC brings the capability to absorb a broader spectrum of solar radiation [3, 4]. Several studies have been conducted on simulation of QDIBSCs. Marti et al. characterized the basic principle of QDIBSC [5]. Ankhi et al. analyzed the InGaAs/GaAs QDIBSC with short-circuit current density (*J*_sc_) of 38.04 mA/cm^2^ [4]. Robichaud et al. predicted the InGaN/GaN QDIBSC with 0.44% conversion efficiency [6]. Rocha et al. simulated the InAsP/InGaP QDIBSC with 1.2 eV transition energy from valance band (VB) to the IB [7].

The design of Ge/Si QDIBSC has also been discussed. In 2013, a group of researchers developed a top-down process to fabricate a type-II Ge/Si QD for use in all Si-based IBSC applications. The theoretical calculation revealed that with many Ge/Si QDs well-aligned and closely packed, their wave functions diffuse into neighbors and couple with each other, forming quasi-continuous minibands. So the Ge/Si QD superlattice (SL) was inserted into the Si p–n junction and induced an additional two photon transition, which increased photocurrent [8]. In 2016, a 3D finite element method was employed to compute the miniband structure and density of state (DOS) for the Ge/Si QD SL thorough a numerical simulation. The results proved that, with the various dimension and square SL of QDs, the formed miniband works as IB that can absorb sub-bandgap photons to increase the *J*_sc_ [9]. In 2017, an IBSC based on Ge/Si QD SL was designed. In that work, the Schrödinger equation was solved by the finite element method to compute the miniband structure of multilayer QDs array. The photocurrent characteristics of IBSC were calculated using the Luque theory for different geometries of QD and a high conversion efficiency of 27.22% was observed [10].

According to recent studies, graphene has been widely exploited to enhance the performance of the thermionic and dye-sensitized solar cells [11–13]. Due to unique properties and quantum confinement of graphene, graphene-based QDs provide a means to create a sub-bandgap in the energy band structure of Si. The high edge/bulk ratio of graphene edges has great influence on the properties of graphene nanoconstraint structures, for example, nanoribbons and quantum dots. However, the absence of a bandgap in graphene limits its incorporation in optoelectronic devices. The interaction between graphene and a Si surface is strong, leading to formation of chemical bonds and a large bandgap. Recently, researchers interfaced graphene with silicon to form Schottky junctions that are useful in photo diodes, light harvesters and solar cells [14–16]. In this paper, we propose a new multilayer graphene/Si QD SL structure to create IB for Si-based solar cells. We implement our simulation with graphene as the unique candidate to design the QD core. For design of the shell, we focus on Si, because Si-based solar cells hold the world record in efficiency and can benefit from a well-developed manufacturing process [17]. After designing the graphene/Si QD structure, we create a SL of these QDs and obtain its band structure as well as energy states based on the Slater–Koster (SK) tight-banding (TB) method using Atomistix-Toolkit (ATK) software. Next, the energy level hybridization influenced by the inter-dot spacing among QDs in the SL is investigated. Finally, to identify the impact of the graphene/Si QD SL on the performance of the Si-based solar cell, through creating the IB, we design a graphene/Si QDIBSC and calculate its characteristics using Lumerical software. By comparing the results with and without quantum dots, we observed improvement of the properties of solar cell with the graphene/Si QD SL included.

## Material and methods

### Structure of graphene/Si QD superlattice

This section describes the design of the graphene/Si QD. To simulate the graphene/Si QD, we first create a (12,0) Si–Si nanotube with 0.7 Å bond distance as the QD shell. Then to design the QD core, we use the multilayer graphene (MLG) sheets with AA stacking and 0.3-nm distance between graphene layers. In this way, each carbon atom of the second layer is placed exactly above the corresponding atom of the first carbon layer. Next, an interface of MLG (core) and Si nanotube (shell) is constructed. Finally, we shift the shell toward the core, so that MLG is surrounded by Si nanotube. Each Si atom pushes the nearest carbon neighbors away, inducing distortion in the local area. Si atoms at the graphene edge prefer *sp*^2^ hybridization, bonded with four carbon atoms. We delete the carbon atoms which are located outside of the shell. The graphene layers form strong bonds with bare Si via covalent bonding to Si.

**Fig. 1:**
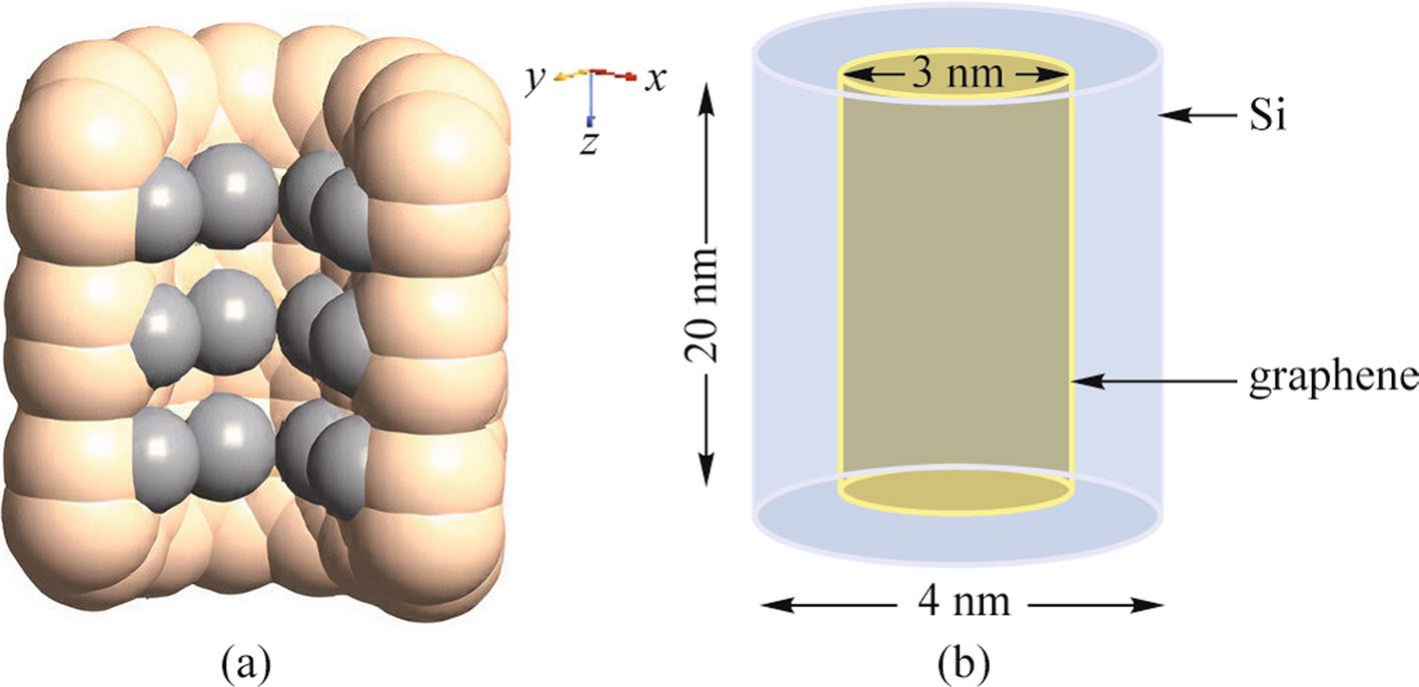
**a** Atomistic structure of the graphene/Si QD; **b** schematic structure of the graphene/Si QD

The developed interaction pulls the carbon atoms in graphene layers towards the silicon atoms and creates a stable bonding between them. Despite strong Si–C bonds there are no interactions between graphene layers. The weak Van der Waals interlayer coupling in graphene multilayers exert a significant influence on the energy levels, leading to new properties. MLG structure presents an electrical bandgap derived from intrinsic optical bandgap generated by the confinement of carriers and the biased electrical field, mainly due to weak anti-localization behavior of carriers [18, 19]. Figure 1 shows the atomistic and schematic structure of the graphene/Si QD.

Several methods have been developed to fabricate graphene/Si structures. Earlier studies showed formation of graphene on silicon by annealing epitaxial SiC film grown on silicon substrate at 1500 K [20]. Transfer of graphene on silicon (111) surface under ultra-high vacuum conditions by fusion bonding or wafer direct bonding has been discussed [21]. Laser beam induced growth of graphene on silicon is another technique [15]. Molecular beam epitaxy (MBE) has been used to assemble the periodic graphene/Si structure layer by layer. Intercalation, where the deposited Si atoms do not stay at the graphene surface but surround graphene layers, may be happen at room temperature [22]. According to our design process, it seems that intercalation may be an appropriate method for fabrication of this type of graphene/Si QD.

**Fig. 2:**
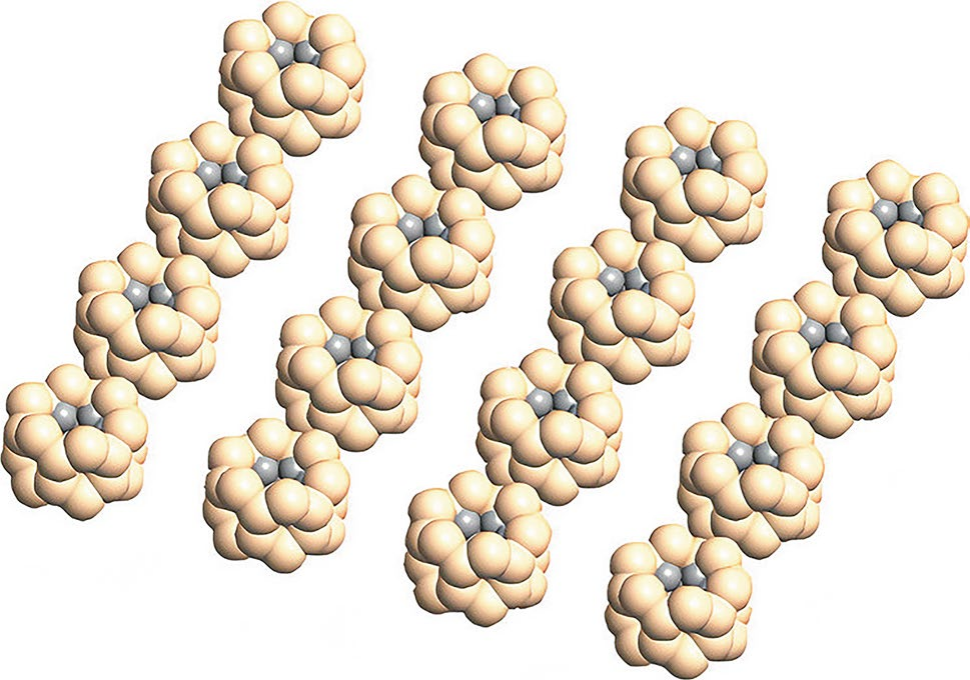
Graphene/Si QD super lattice

A single QD is not able to make a mini-band. The question is that how many neighbors should be included in the Hamiltonian? According to the previous studies, including only the first neighbors is not enough for the mini-band creation, the inclusion of second and often the third neighbors is necessary. As a result, the SL is designed by involving three neighbors as is shown in Fig. 2. The energy level hybridization has a direct relation with the inter-dot spacing among QDs in the SL.

#### Method for computing the electronic structure of the graphene/Si QD

In general, it is difficult to simulate electronic devices in which quantum effects play an important role. Due to advances in nanotechnology, dimensions of electronic devices have been shrunk down to nanoscale. As electrons are trapped in nanoscale regions, the electronic states can be quantized and their behavior can only be described by the Schrödinger equation. The finite-difference time-domain (FDTD) method has been employed in modeling metamaterials and conventional Maxwell–Schrödinger systems with electromagnetic fields [23].

For calculating the electronic band structure of the graphene/Si QD, the SK-TB method is performed using ATK software. The SK-TB method or linear combination of atomic orbitals (LCAO) is a semi-empirical method that is primarily used to calculate the band structure and single-particle Bloch states of a material. For large systems containing up to hundreds of atoms, the density function theory (DFT) is used to find the true ground state density and ground state energy of an interaction system without explicitly calculating the many-electron wave function. A unit cell that describes the periodicity of SL is defined to reduce the sampling size of *k*-points for simulation. The unit cell includes 18 carbon atoms and 144 Si atoms with *sp*^2^ hybridization, where reformations such as elimination and shifting some atoms are applied to an initial design in order to obtain the basic structure as is shown in Fig. 1a. A $10\times 10$ *k*-point sampling grid, cutoff energy of 160 Ry, the Hamiltonian matrix elements that are related to the orbitals that participate in bonding between atoms, and periodic boundary conditions are used to perform these calculations. The SK-TB formalism is an extension of Bloch’s original method that shows all of the correct symmetry properties of the energy bands and provides a solution for the single-particle Schrödinger equation at each sampling *k-*points in the irreducible Brillouin zone (IBZ) [24–26]. Equation (1) represents the Schrödinger equation:

1 $$\left[-\frac{{h}^{2}}{2m}{\nabla }^{2}{\Psi }_{i}\left(\overrightarrow{r}\right)+V\left(\overrightarrow{r}\right){\Psi }_{i}\left(\overrightarrow{r}\right)\right]={E}_{i}{\Psi }_{i}\left(\overrightarrow{r}\right),$$

where *h*, *m*, *V*, *Ψ*, and *E* are the Plank constant, electron mass, potential energy, the wave function, and eigenvalues of energy, respectively. Wave functions are approximated by the combination of Bloch functions. The electron wave functions can be obtained through:

2 $${\Psi }_{i}\left(\overrightarrow{k},\overrightarrow{r}\right)=\sum_{j=1}^{n}{C}_{i,j}\left(\overrightarrow{k}\right){\mathrm{\Phi}}_{j}\left(\overrightarrow{k},\overrightarrow{r}\right) ,$$

where $\overrightarrow{k}$, *C*, and Ф are wave vectors, weight coefficients, and Bloch function, respectively. If there are *n* wave vectors considered in every unit cell and there are *N* unit cells in crystal lattice, whose position are denoted by $\overrightarrow{R}$, then the Bloch functions will be as Eq. (3):

3 $${\mathrm{\Phi}}_{j}\left(\overrightarrow{k},\overrightarrow{r}\right)=\frac{1}{\sqrt{N}}\sum_{l=1}^{N}{\mathrm{e}}^{\text{i}\overrightarrow{k}\cdot\overrightarrow{{r}_{l}}}{\mathrm{\Phi}}_{j}\left(\overrightarrow{r}-\overrightarrow{{R}_{l}}\right).$$

For finding the weight coefficients, the values of *E* should become optimum through Eqs. (4) and (5):

4 $${E}_{i}\left(\overrightarrow{k}\right)=\frac{\int {\Psi }_{i}^{*}H{\Psi }_{i }\mathrm{d}\overrightarrow{r}}{{\Psi }_{i}^{*}{\Psi }_{i}\mathrm{d}\overrightarrow{r}} ,$$

5 $$\left[H\right]{C}_{r}={E}_{i}\left(\overrightarrow{k}\right)\left[S\right]{C}_{i} ,$$

where *H* and *S* are the Hamiltonian and overlap matrixes. The overlap matrix can be obtained through:

6 $${S}_{ij}=\langle {\mathrm{\Phi}}_{i}|{\mathrm{\Phi}}_{j}\rangle .$$

#### Method for simulating graphene/Si QDIBSC

**Fig. 3:**
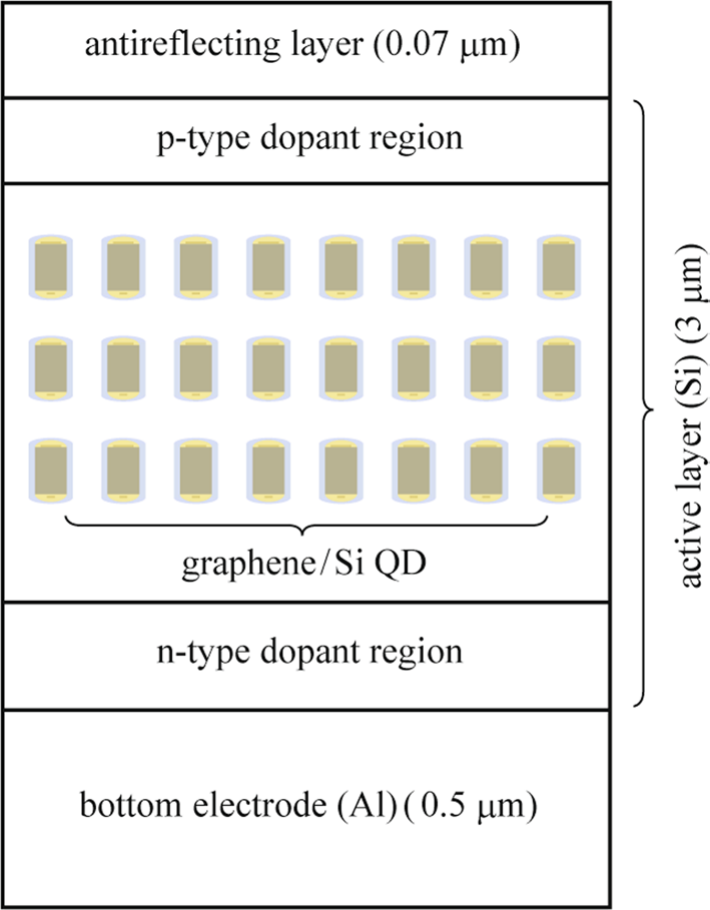
Base structure of graphene/Si QDIBSC

To evaluate the impact of graphene/Si QD SL on the performance of the Si-based solar cell, we design and simulate a graphene/Si QDIBSC. We obtain the short-circuit current density (*J*_sc_) and electron–hole generation rate (*G*) using Lumerical software. Figure 3 presents the structure of the graphene/Si QDIBSC. This cell consists of three layers: the anti-reflection (AR) layer with 0.07 µm thickness and refractive index of 2.05, the Si layer with 3 µm thickness as the active layer, and the Al layer with 0.5 µm thickness as the bottom electrode. A three-layer closely packed SL of the graphene/Si QDs is embedded in the active layer. In the simulation, the solar cell structures are illuminated by a light source with a spectral range of 0.3–5 µm along the direction of − *Y*. The periodic and PML boundary conditions are chosen in *X* and *Y* directions, respectively. Using nanostructures such as QDs often causes an open-circuit voltage drop which is observed when sub-bandgap states are introduced [27]. This drop denotes strong Auger recombination. In addition, the nonradiative recombination associated with the sub-bandgap between IB and CB is expected to be large. So the nonradiative recombination is considered in the simulation process. A p-type region with $2\times {10}^{20}{\mathrm{cm}}^{-3}$ doping concentration and an n-type region with $1\times {10}^{19}{\mathrm{cm}}^{-3}$ doping concentration are taken into account. In between, IB is formed in the bandgap of silicon due to the embedded graphene/Si QDs SL. The solar generation rate analyzer, a functional module provided by Lumerical, is exploited to compute the electron–hole generation rate.

To investigate the characteristics (*J*_sc_ and *G*) of both the Si-based solar cell and the graphene/Si QDIBSC, the solar generation rate analyzer is exploited. This analyzer consists of the Poisson’s equation, the carrier continuity equation for electrons in the CB and holes in VB, and the transport equations in drift–diffusion model. Poisson’s equation relates variations in the electrostatic potential to local charge densities. The continuity and the transport equations describe the way that the electron and hole densities evolve as a result of transport, generation, and recombination processes. In the case of IBSC, there are electrons in IB. The electrostatic potential can be given by the Poisson equation [28–30].

7 $$\frac{{\rm{d}}^{2} \emptyset (y)}{{\rm{d}}y^{2}}=\frac{\varepsilon }{e}\left({n}_{\rm{c}}\left(y\right)-p\left(y\right)+{n}_{1}\left(y\right)-{N}_{\rm{D}}^{+}\left(y\right)+{N}_{\rm{A}}^{-}\left(y\right)\right)n,$$

where *y* is the direction of light emission and the along of the electrical field distribution, *e* is the elementary charge, *ε *is the dielectric constant of the material, *p*(*y*) is the hole density, *n*_c_(*y*) is electron density in CB, *n*_1_(*y*) is electron density in IB, ${N}_{\mathrm{D}}^{+}(y)$ is the ionized donor density, and ${N}_{\mathrm{A}}^{-}(y)$ is the ionized acceptor density. In this work, acceptors and donors are assumed completely ionized, i.e., ${N}_{\mathrm{D}}^{+}(y)$ = ${N}_{\mathrm{D}}(y)$ and ${N}_{\mathrm{A}}^{-}(y)$=${N}_{\mathrm{A}}(y)$, where ${N}_{\mathrm{D}}(y)$ and ${N}_{\mathrm{A}}(y)$ are the donor and the acceptor densities, respectively. In the steady-state, these carriers satisfy carrier continuity equations for electrons in CB (Eq. (8)) and holes in VB (Eq. (9)).

8 $${G}_{\mathrm{cv}}\left(y\right)-{U}_{\mathrm{cv}}\left(y\right)+{G}_{\mathrm{ci}}\left(y\right)-{U}_{\mathrm{ci}}\left(y\right)+\frac{1}{e}\frac{\mathrm{d}{J}_{\mathrm{c}}\left(y\right)}{\mathrm{d}y}=0,$$

9 $${G}_{\mathrm{cv}}\left(y\right)-{U}_{\mathrm{cv}}\left(y\right)+{G}_{\mathrm{iv}}\left(y\right)-{U}_{\mathrm{iv}}\left(y\right)+\frac{1}{e}\frac{{\mathrm{d}J}_{\mathrm{v}}\left(y\right)}{\mathrm{d}y}=0,$$

where *G*_*ij*_ is the optical generation rate, *U*_*ij*_ is the radiative recombination rate where subscript *ij* = ci, iv, and cv expresses CB-IB, IB-VB, and CB-VB transition respectively. *J*_c_ is the electron current density in CB and *J*_v_ is the hole current density in VB. *J*_c_ and *J*_v_ are given by drift–diffusion equations shown below:

10 $${J}_{\mathrm{c}}\left(y\right)=-e{\mu }_{\mathrm{e}}{n}_{\mathrm{c}}\left(y\right)\frac{\rm{d} \emptyset ({y})}{\mathrm{d}y}+e{D}_{\mathrm{e}}\frac{\mathrm{d}{n}_{\mathrm{c}}\left(y\right)}{\mathrm{d}y},$$

11 $${J}_{\mathrm{v}}\left(y\right)=-e{\mu }_{\mathrm{h}}p\left(y\right)\frac{\rm{d} \emptyset(y)}{\mathrm{d}y}-e{D}_{\mathrm{h}}\frac{\mathrm{d}p\left(y\right)}{\mathrm{d}y},$$

where *μ*_e_ and *μ*_h_ are the carrier mobility of electrons in CB and holes in VB, *D*_e_ and *D*_h_ are the diffusion coefficients, respectively.

## Results

### Electronic structure of the graphene/Si QD

**Fig. 4:**
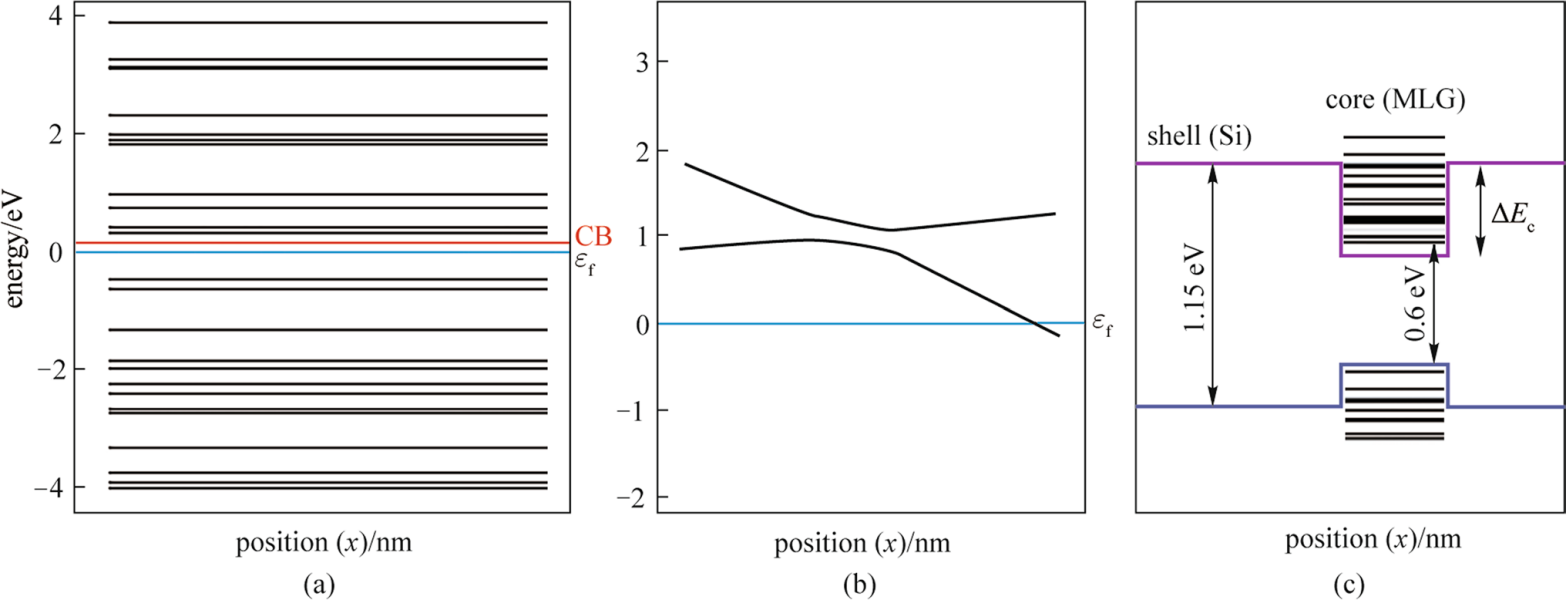
**a** Bound state energy levels for the single graphene/Si QD; **b** effect of the strain on the energy levels of graphene/Si QD; **c** calculated band alignment for the graphene/Si QD

Figure 4a shows the discrete energy levels of the graphene/Si QD. The states that have lower energies than the CB band edge of the host material are called the bound states and the states with higher energies than the CB edge of host material are called virtual bound states. The bound states are usually named by the quantum numbers in the *x*, *y*, and *z* dimensions, respectively, e.g., (1,1,1) and (2,2,1). The bound state with the quantum number (1,1,1) is called the ground state and it constitutes the IB [31]. The ground state in the graphene/Si QD is 0.6 eV above the VB. It is important to have the IB energy level at 1/3 or 2/3 of the bandgap for equalizing IB photocurrents [31]. As the bandgap of graphene is zero, and the effective bandgap of graphene/Si QD decreases as the number of graphene layers increases, the number of graphene layers must not exceed 3. The interaction at the interface of Si and graphene creates band offsets in VB and CB and introduces strain near the interface. Strain induces a shift in the energy levels. Figure 4b depicts a localized shift of energy levels of the graphene/Si QD under strain. Figure 4c demonstrates energy band alignment for the graphene/Si QD when the designed geometry parameters (4 nm outer-diameter, 3 nm inner-diameter, and 20 nm height) are used. It exhibits a typical type-I quantum dot structure. The blue line is VB edge, the violet line is CB edge and the black lines are the quantized energy levels for CB and VB electrons inside QD. The value of the band offset for CB (Δ*E*_c_) is 0.33 eV.

**Fig. 5:**
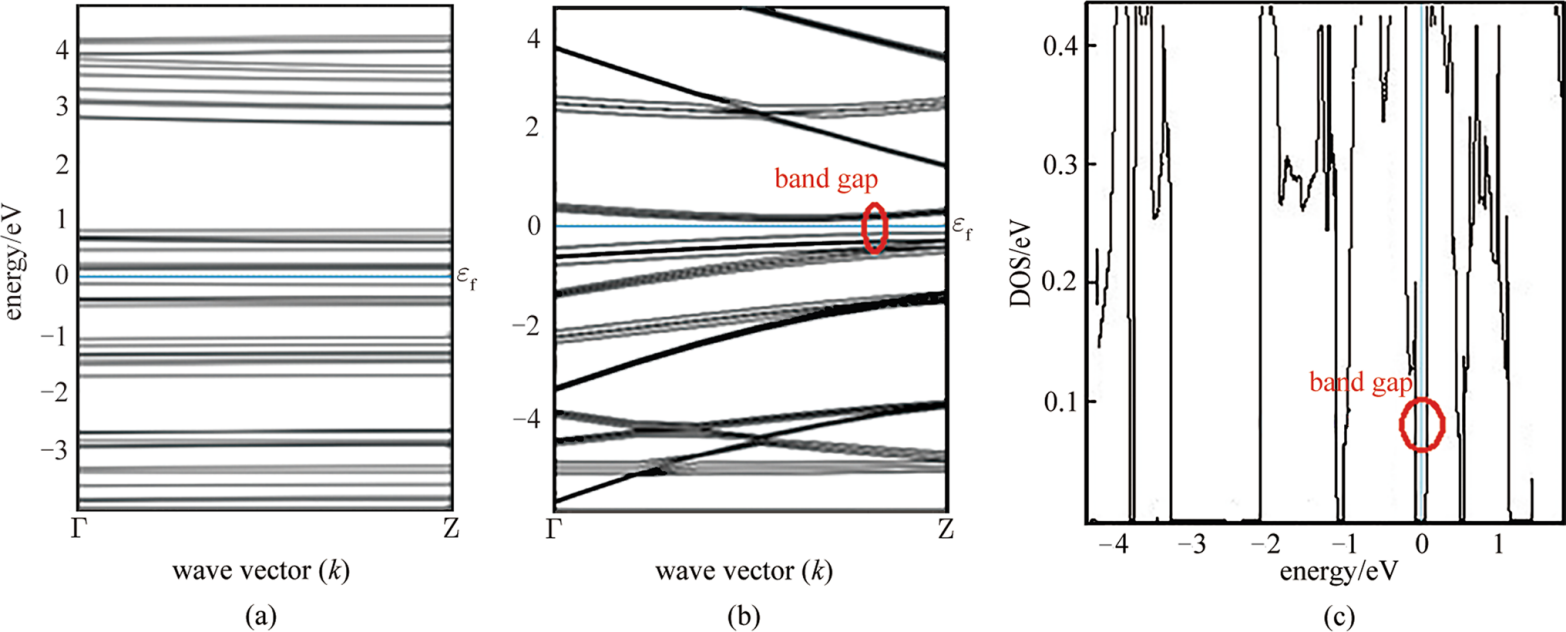
Band structure in *k* space along wave vector for the graphene/Si QDs SL; **a** with an 8 nm inter-dot spacing; **b** for closely packed SL; **c** density of states for closely packed graphene/Si QDs SL

Figure 5 demonstrates the band dispersion relation of graphene/Si QDs for different inter-dot spacings. For the band dispersion calculation, *k* varies along the edges of first IBZ. As shown in Fig. 5a, when inter-dot spacing is 8 nm, there is no interaction among QDs and the SL behaves like a single QD. If the inter-dot spacing decreases, the density of QDs increases and then their wave functions spread into neighbors and couple with each other, resulting in the formation of the mini-band. The mini-band formation phenomenon in a well-aligned and closely packed SL is shown in Fig. 5b. Figure 5c shows the density of state (DOS) for graphene/Si QDs SL with a bandgap of roughly 0.3 eV.

### Characteristics of Si-based solar cell with and without graphene/Si QD layers

**Fig. 6:**
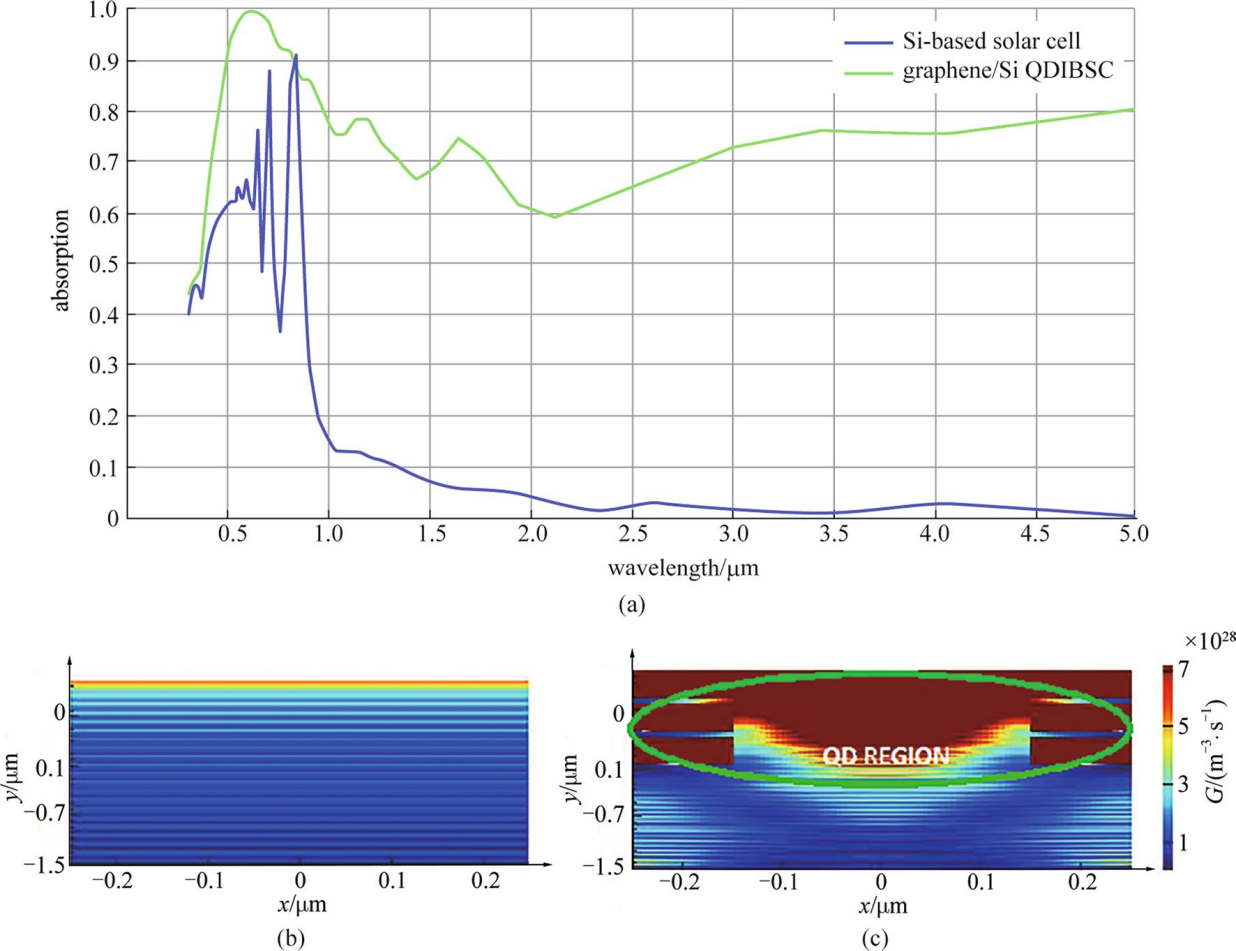
**a** Absorption of the Si-based solar cell with active layer thickness of 3 µm and Graphene/Si QDIBSC; **b** generation rate for Si-based solar cell; **c** generation rate for Graphene/Si QDIBSC

A Si-based solar cell with active layer thickness of 3 µm is simulated under illumination of a light source with spectral range of 0.3–5 µm. The *J*_sc_ and *G* are estimated to be 16.9067 mA/cm^2^ and 1.48943 × 10^28^ m^−3^·s^−1^ for Si-based cell without AR layer and 21.1465 mA/cm^2^ and 3.16059 × 10^28^ m^−3^·s^−1^ for Si-based cell with AR layer, respectively. It is clear that the active layer with thickness of 3 µm has small light absorption. So the graphene/Si QD layers are embedded in the active layer of the Si-based solar cell in the simulation. Graphene/Si QD SL introduce intermediate states which enhance absorption of Si layer as is shown in Fig. 6a. The graphene/Si QDIBSC records a considerable improvement with *J*_sc_ = 24.5192 mA/cm^2^ and *G* = 7.79295 × 10^28^ m^−3^·s^−1^ when one layer of QD is embedded. Figure 6b and c compare the generation rate of Si-based solar cell without and with 3 layers of QDs. The material parameters and model geometry that are used in simulation are presented in Table 1.

**Table 1:** Model geometry and material parameters used in the simulation

Layer	Material	Thickness /µm	Refractive index	Electron mobility /(cm^2^ ·V^−1^·s^−1^)	Hole mobility /(cm^2^ ·V^−1^·s^−1^)	Work function /eV	Electron affinity /eV	Dielectric constant	Lattice constant /Å
AR	–	0.07	2.05	–	–	–	–	–	–
Active	Si	3	–	1471	470	4.59	4.17	-	5.4306
Electrode	Al	0.5	–	–	–	–	–	–	–
QD SL	Graphene/Si	0.02	–	–	–	4.55	–	–	–
–	Graphene	–	–	–	–	–	4.5	5.6053	2.4612

When QD SL is embedded in the active layer of the Si-based solar cell, *G* increases significantly. It means that the IB created by the graphene/Si QDs SL, splits the total bandgap of active layer (*E*_G_) into two sub-bandgaps. These sub-bandgaps absorb photons with energy less than *E*_G_. This process allows for increased *G* via a two-step photon absorption from the VB to the IB and from the IB to the CB. The absorption of sub-bandgap photons results in an additional photocurrent. The embedded graphene/Si quantum dots indeed help increase the photo-generated current as expected and thus improve the conversion efficiency of solar cell.

The number of QD layers and shape of QDs strongly influence the quantized energy levels and optical transitions [32]. In order to understand the effect of heights of QD (*H*) and the number of QD layers (*L*) on the graphene/Si QDIBSC performance, we design and simulate graphene/Si QDIBSC with different *H* and *L*. The characteristics of *J*_sc_ and *G* versus *L* (when *H* is fixed at 20 nm) and *H* (when *L* is fixed at 2) are reported in Tables 2 and 3, respectively.

**Table 2:** Performance of the graphene/Si QDIBSC versus *L*

*L* (number)	*J*_sc_/(mA·cm^−2^)	*G*/(m^−3^·s^−1^)
0	21.1468	3.16059 × 10^28^
1	24.5192	7.79295 × 10^28^
2	36.4193	7.94192 × 10^28^
3	35.6109	7.20089 × 10^28^
4	32.8164	7.05904 × 10^28^

**Table 3:** Performance of the graphene/Si QDIBSC versus *H*

*H*/nm	*J*_sc_/(mA·cm^−2^)	*G*/(m^−3^·s^−1^)
16	32.8298	7.12128 × 10^28^
20	36.4193	7.94192 × 10^28^
24	39.131	7.43087 × 10^28^
28	36.7065	7.24695 × 10^28^

As the more layers are embedded higher *J*_sc_ is achieved. Because as *L* increases, more bound states are introduced by additional QD layers. However, according to the results given in Table 2, there is no improvement in the performance of the QDIBSC for *L* $>2.$

When two QD layers are embedded in the active layer of QDIBSC, we alter *H*. As *H* increases, *J*_sc_ also increases. However, for *H* $>24 \text{ nm}$, it seems that *J*_sc_ becomes saturated due to overlapping of minibands. Because we fix the position of QD layers, when we increase *H,* the spacing between QD layers decreases. As a result, instead of formation of IB, the minibands, which are introduced by two QD layers, overlap.

## Conclusion

A new type-I multilayer graphene/Si QD SL structure for IBSC applications was designed. Its ground energy state was calculated to be 0.6 eV above VB using the SK-TB method. This value of ground energy state provides a means to create an IB in the energy band structure of Si. The mini-band formation in a well-aligned and closely packed SL was observed. Then a Si-based solar cell was simulated and a closely packed SL of QDs was embedded in the active layer of this cell. The *J*_sc_ and *G* of this solar cell with and without QD SL were obtained by applying the 2D-FDTD method under illumination of a light source with spectral range of 0.3–5 µm. When the QD SL including one layer QD is embedded *G* was roughly tripled. *J*_sc_ was increased approximately 16% from 21.1468 to 24.5192 mA/cm^2^. Finally the dependence of IBSC performance on the important factors of QD SL, including *L* and *H* was investigated. For number of QD layers *L* $>2,$ there was no significant improvement in the performance of the QDIBSC. For height of QD *H* $>24 \text{ nm},$ the graphene/Si QDIBSC could not reach a higher performance due to the overlapping of minibands**.**
